# A Successful Living Donor Liver Transplantation Using Hepatic Iron Deposition Graft Suspected by Magnetic Resonance Imaging

**DOI:** 10.1155/2023/9075184

**Published:** 2023-03-16

**Authors:** Nobuhiko Kurata, Masato Shizuku, Kanta Jobara, Yoji Ishizu, Masatoshi Ishigami, Yasuhiro Ogura

**Affiliations:** ^1^Department of Transplantation Surgery, Nagoya University Hospital, Nagoya, Aichi, Japan; ^2^Department of Gastroenterology and Hepatology, Nagoya University Graduate School of Medicine, Nagoya, Aichi, Japan

## Abstract

Recently, magnetic resonance imaging (MRI) has been developed as a widely available and noninvasive method for detecting and evaluating hepatic iron overload. This case report presents a successful living donor liver transplantation (LDLT) in which the donor was suspected to have hepatic iron deposition by MRI evaluation. A preoperative donor liver biopsy and genetic examination were performed to exclude hereditary hemochromatosis and other chronic liver diseases. A liver biopsy showed an almost normal liver specimen with a slight deposition of iron in 2-3% of hepatocytes, and a genetic examination of hereditary hemochromatosis revealed no typical mutations in HFE, TFR2, HJV, HAMP, or SLC40A1. Despite the traumatic hemothorax complication caused by the liver biopsy, the liver transplant eligibility was confirmed. Two months after the hemothorax complication, an LDLT donor operation was performed. The donor was discharged from the hospital on postoperative day (POD) #17 with favorable liver function. The recipient's posttransplant clinical course was generally favorable except for acute cellular rejection and biliary complications, and the recipient was discharged from the hospital on POD #87 with excellent graft function. A one-year follow-up liver biopsy of the recipient demonstrated almost normal liver with iron deposition in less than 1% of the hepatocytes, and no iron deposition was identified in the liver graft by MRI examination. Liver biopsy and genetic examination are effective methods to evaluate the eligibility of liver transplant donors with suspected hepatic iron deposition. The living donor with slight hepatic iron deposition, if hereditary hemochromatosis was ruled out, can donate partial liver safely.

## 1. Introduction

Iron is an essential mineral in the body and plays an important role in cellular and organismal processes such as heme synthesis, DNA synthesis, and oxidative phosphorylation. While iron deficiency induces anemia, excess iron produces free oxygen radicals that can be toxic to DNA, proteins, and lipids [1, 2]. As the human body has no physiological method to excrete iron, iron regulation is tightly controlled by the absorption from dietary sources in the duodenum and proximal jejunum, recycling of iron in erythrocytes by macrophages, and body storage [3].

It has been shown that the liver is the major storage site for iron and plays a vital role in iron homeostasis. Several genes related to hepatic iron regulation have been identified, including HFE, HJV, and HAMP [1–3]. If the living donor has a hereditary disease of iron hemostasis, the donation of partial liver is considered unsuitable. Moreover, previous reports regarding the liver biopsy from healthy potential living liver donors demonstrated that the presence of iron deposits in the liver was associated with steatohepatitis, which could be a contraindication for partial liver donation [4]. Hepatic iron deposition is caused not only by hereditary hemochromatosis and other chronic liver diseases but also by multiple blood transfusions, drugs, and oral nutritional supplementation [5, 6]. Therefore, we must be extremely careful to evaluate the living donor candidate with possible iron deposition.

Magnetic resonance imaging- (MRI-) based liver susceptibility measurement techniques have recently been developed as widely available and noninvasive methods for detecting and evaluating iron overload in the liver [7–9]. In this case report, we present a successful living donor liver transplantation (LDLT) in which preoperative donor liver biopsy and genetic examination were performed to confirm the eligibility of the liver graft since the donor was suspected to have hepatic iron deposition by MRI evaluation.

## 2. Case Presentation

A 31-year-old Japanese man, who was diagnosed with primary sclerosing cholangitis and ulcerative colitis at the age of 17, was referred to our transplant center. The patient presented with severe jaundice and was suffering from a recurrent episode of bacterial cholangitis. Contrast-enhanced computed tomography revealed an enlarged liver with an irregular surface, splenomegaly, massive ascites, esophageal varices, and splenorenal shunt. His Child-Pugh score was 10 (grade C), and the model for end-stage liver disease score was 20. Although preparation of living donor as well as cadaveric liver transplantation was initiated, preparation of living donor liver transplantation was accelerated because of consideration of the waiting time of deceased donor liver transplantation in our country.

**Figure 1:**
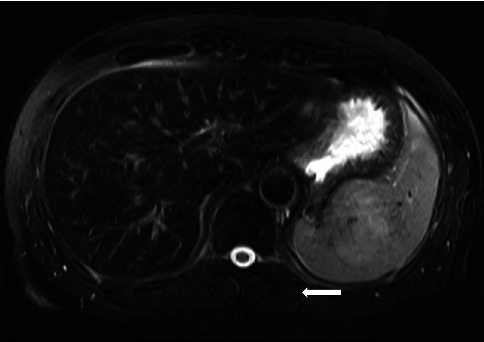
Axial T2-weighted magnetic resonance image demonstrates that the signal intensity of the liver is lower than that of the paraspinal musculature (white arrow), indicating hepatic iron deposition.

The evaluation of the patient's father as a potential donor for LDLT was initiated. Although contrast-enhanced computed tomography showed normal liver appearance, bowel wall thickening of the sigmoid colon was noticed despite of no physical symptoms. A 25-mm polyp in the sigmoid colon was detected on colonoscopy, and endoscopic mucosal resection was performed. Because the pathological examination revealed carcinoma in adenoma and curative resection was already achieved, we considered this donor candidate as applicable for hepatic donation. Further examination proceeded, then laboratory investigations showed normal liver function, but iron-related tests showed some abnormalities. Although serum iron, transferrin, and total iron-binding capacity levels were within normal limits (54 *μ*g/dL, 202 mg/dL, and 273 *μ*g/dL, respectively), we found mild elevation of ferritin level (453 ng/mL, normal range 22-­275). In magnetic resonance cholangiopancreatography for biliary tree evaluation, radiologists pointed out mild hepatic iron deposition due to lower signal intensity of the liver compared to the signal of paraspinal musculature on T2-weighted images (Figure 1). After the identification of iron-related abnormalities, we interviewed him again about any kinds of physical issues or foods/supplements, etc. However, he had no history of blood transfusion, regular medication use, or oral nutritional supplement intake. Percutaneous liver biopsy and further genetic testing were performed because hereditary hemochromatosis and other chronic liver diseases could not be excluded.

**Figure 2:**
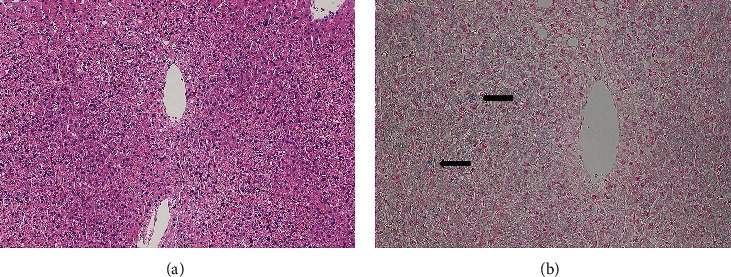
Preoperative donor percutaneous liver biopsy showed an almost normal liver with slight deposition of iron in 2-3% of hepatocytes (arrows in B), but not in the Kupffer cells. (a) Hematoxylin and eosin stain. (b) Berlin blue stain.

Liver biopsy showed an almost normal liver with a slight deposition of iron in 2-3% of hepatocytes, but no iron deposition in the Kupffer cells (Figures 2(a) and 2(b)). Unfortunately, he developed traumatic hemothorax after liver biopsy, which required thoracic drainage.

Genetic examination revealed no mutations in HFE, TFR2, HJV, HAMP, or SLC40A1, which are representative of hereditary hemochromatosis and ferroportin disease. The level of hepcidin-25 (82.9 ng/mL), which is the principal regulator of iron absorption and may be high in ferroportin disease, was slightly elevated, but it could be considered normal range under the elevated level of serum ferritin [1, 10]. Ferroportin disease was ruled out because of hepcidin-25 level, no mutation in SLC40A1 in the genetic test, and no Kupffer cell proliferation in the liver biopsy (Table 1). Aceruloplasminemia, which is a rare genetic disorder characterized by the abnormal accumulation of iron in the brain and various internal organs, was also ruled out because the ceruloplasmin level of 20 mg/dL was almost in normal level (normal range 21-37). Based on these results, we excluded hereditary hemochromatosis and other chronic liver diseases and finally decided to proceed with donor operation two months after recovery of hemothorax.

**Figure 3:**
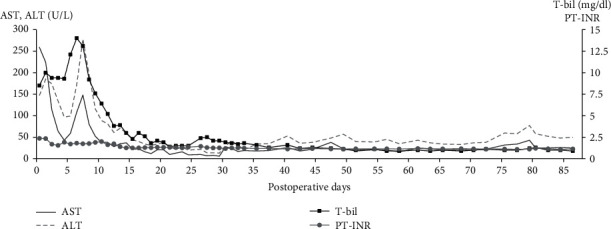
Postoperative clinical course of the LDLT recipient. Abbreviations: AST: aspartate aminotransferase; ALT: alanine aminotransferase; PT-INR: prothrombin time-international normalized ratio; T-bil: total bilirubin.

He donated the right lobe graft weighing 874 g, and the remnant liver was estimated at approximately 30.2%. The liver function test recovery was relatively slow probably due to a smaller remnant liver volume. The donor was discharged from the hospital on POD #17 with favorable liver function. The recipient's posttransplant clinical course and graft function were generally favorable including iron homeostasis, although the recipient required treatment for acute cellular rejection and biliary complications. The recipient was discharged from the hospital on POD #87 (Figure 3).

**Figure 4:**
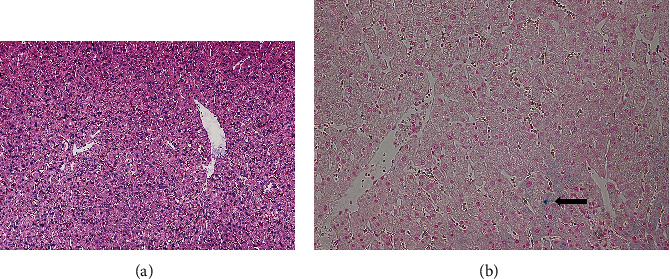
The recipient's percutaneous liver biopsy one year after liver transplantation showed an almost normal liver with iron deposition in less than 1% of the hepatocytes (arrow in B). (a) Hematoxylin and eosin stain. (b) Berlin blue stain.

The recipient's percutaneous liver biopsy one year after LDLT showed an almost normal liver with iron deposition in less than 1% of the hepatocytes (Figures 4(a) and 4(b)). There was no hepatic iron deposition in the liver graft identified on MRI one year after LDLT.

## 3. Discussion

As the living donor liver transplantation, any kinds of concerns must be cleared. During the living donor assessment process in this case, we noticed a slight difference in MRI findings. To answer this question, liver biopsy and genetic examination were added to check his iron metabolism.

Iron is an essential element in human cell function, particularly for hemoglobin and myoglobin production. Because it is difficult to excrete excessive iron from the human body, iron homeostasis is tightly controlled to avoid the toxicity of free oxygen radicals that are generated by systemic iron overload [1–3]. Hepcidin is a hormone synthesized in the liver that negatively regulates the iron absorption of enterocytes and is exported to the plasma by macrophages. Hereditary hemochromatosis is defined as pathological hepatic iron overload caused by hepcidin deficiency [1]. Other than hereditary hemochromatosis, hepatic iron deposition is known to occur in chronic liver diseases, such as alcoholic cirrhosis, chronic viral hepatitis, and nonalcoholic fatty liver disease. Moreover, secondary iron overload can also occur in several conditions, including multiple blood transfusions, long-standing dialysis, and anemias with ineffective erythropoiesis (Table 2) [3, 5].

In the present case, the donor was suspected to have a hepatic iron overload on our routine evaluation. MRI is a widely accepted method for detecting and quantifying hepatic iron overload [7–9]; however, the notification of hepatic iron overload by magnetic resonance cholangiopancreatography for our LDLT donor evaluation was rare. The MRI finding suggested a need for further examinations to rule out hereditary etiologies that should be a contraindication for liver transplant donation according to the decision algorithm described in the previous report [11]. We performed a liver biopsy and genetic examination to investigate whether the donor candidate had liver transplant eligibility. Hereditary hemochromatosis was excluded by the absence of mutations in genetic studies. High hepcidin levels were in accordance with only type 4 hereditary hemochromatosis (ferroportin disease), but ferroportin disease was ruled out because there was no mutation in SLC40A1 and no iron deposition in the Kupffer cells in the liver biopsy [2]. Liver biopsy showed only a slight iron deposition in hepatocytes and no findings of chronic liver disease as well as hepatic fibrosis. We concluded that this donor candidate was eligible for LDLT donation based on the results of multifactorial analysis including the liver biopsy and genetic examination. The immediate postoperative clinical courses in both donor and recipient were favorable, and liver function was excellent at the outpatient clinic.

The donor developed traumatic hemothorax after the liver biopsy and required thoracic drainage. Previous reports have shown that LDLT donor screening liver biopsy was useful with a low complication rate and provided valuable information to increase donor safety while maximizing the donor pool [12–14]. Certainly, liver biopsy was an effective examination in the present case to evaluate the extent of hepatic iron deposition and to exclude other chronic liver diseases [11]. Although MRI examination showed hepatic iron overload, laboratory tests revealed normal liver function. The serum ferritin level of 453 ng/mL was mildly above the upper limits of the normal range (<300 ng/mL in men). Serum ferritin level may exceed to 1000 ng/mL in some cirrhosis patients with hereditary hemochromatosis [15, 16], but our donor candidate did not show such a high level of ferritin. Hereditary hemochromatosis was denied due to no mutations in the genetic examination. It has been reported that liver transplant recipients using a graft with donor iron overload demonstrated similar clinical outcomes compared to normal hepatic iron overload. In these cases, the finding of donor hepatic iron overload could not be recognized at the time of organ procurement because these donors were asymptomatic with normal liver function in the laboratory tests. The 1-hour recirculation wedge biopsy revealed 3+ hemosiderosis by iron stains [17]. Whether a liver biopsy should be performed in the case of normal liver function in laboratory tests and no mutations on genetic tests is an issue that needs further analysis. However, we believe that liver biopsy was necessary in this case to exclude underlying liver diseases both for donor safety and for graft function.

In this case report, we present a successful living donor liver transplantation case after the confirmation of eligibility by liver biopsy and genetic examination in the donor candidate of possible hepatic iron deposition suspected by MRI examination. Liver biopsy and genetic examination are effective methods to evaluate the eligibility of liver transplant donors with suspected hepatic iron deposition. These evaluation steps must be done to avoid donor risks from their underlying iron-related disease and recipient risks related to their poor qualities of iron deposition graft. After these evaluations, the living donor with slight hepatic iron deposition can donate partial liver safely.

## Figures and Tables

**Table 1:** Summary of representative types in hereditary hemochromatosis.

Category	Causative gene	Features
Type 1	HFE	Most common HH category
Type 2A	HJV (hemojuvelin)	Onset at a younger age than the other HH categories; most severe form of systemic iron overload
Type 2B	HAMP (hepcidin)	
Type 3	TFR2	Mutations in transferrin receptor 2; very rare
Type 4	SLC40A1 (ferroportin)	Only HH category with elevated hepcidin level; more iron deposition located in the Kupffer cells than in hepatocytes

HH: hereditary hemochromatosis.

**Table 2:** Possible causes that lead to secondary iron overload.

Chronic liver disease	Chronic hepatitis B or C virus infection
	Nonalcoholic fatty liver disease
	Alcoholic liver disease
	Cirrhosis

Hematological disorders	Thalassemia
	Sickle cell disease
	Hereditary spherocytosis
	Aceruloplasminemia
	Anemia of chronic disease

Other conditions	Multiple blood transfusions
	Long-standing dialysis
	Excess iron intake (dietary/vitamin supplements)

## Data Availability

The datasets generated and/or analyzed during the current study are available from the corresponding author on reasonable request.

## Abbreviations

LDLT: Living donor liver transplantation
MRI: Magnetic resonance imaging
POD: Postoperative day.
